# Evaluation of an accelerometer-based monitor for detecting bed net use and human entry/exit using a machine learning algorithm

**DOI:** 10.1186/s12936-022-04102-z

**Published:** 2022-03-12

**Authors:** Guibehi B. Koudou, April Monroe, Seth R. Irish, Michael Humes, Joseph D. Krezanoski, Hannah Koenker, David Malone, Janet Hemingway, Paul J. Krezanoski

**Affiliations:** 1 Centre Suisse de Recherches Scientifiques en Côte d’Ivoire, 01 BP 1303 Abidjan 01, Côte d’Ivoire; 2 UFR Science de la Nature, Université Nangui Abrogoua, 02 BP 801 Abidjan 02, Côte d’Ivoire; 3 PMI VectorWorks Project, Johns Hopkins Center for Communication Programs, Baltimore, MD USA; 4 US President’s Malaria Initiative, Center for Diseases Control and Prevention (CDC), Atlanta, USA; 5 US President’s Malaria Initiative, United States Agency for International Development (USAID), Washington, DC USA; 6 Opportunity Solutions International, San Francisco, CA 94118 USA; 7 Malaria Department, Bill and Melinda Gates Foundation, Seattle, USA; 8 Vector Biology Department, Liverpool School of Tropical Medicine, Liverpool, UK; 9 University of California, San Francisco, San Francisco, CA USA

**Keywords:** Malaria prevention, Bed net use, Machine learning

## Abstract

**Background:**

Distribution of long-lasting insecticidal bed nets (LLINs) is one of the main control strategies for malaria. Improving malaria prevention programmes requires understanding usage patterns in households receiving LLINs, but there are limits to what standard cross-sectional surveys of self-reported LLIN use can provide. This study was designed to assess the performance of an accelerometer-based approach for measuring a range of LLIN use behaviours as a proof of concept for more granular LLIN-use monitoring over longer time periods.

**Methods:**

This study was carried out under controlled conditions from May to July 2018 in Liverpool, UK. A single accelerometer was affixed to the side panel of an LLIN and participants carried out five LLIN use behaviours: (1) unfurling a net; (2) entering an unfurled net; (3) lying still as if sleeping; (4) exiting from under a net; and, (5) folding up a net. The randomForest package in R, a supervised non-linear classification algorithm, was used to train models on 20-s epochs of tagged accelerometer data. Models were compared in a validation dataset using overall accuracy, sensitivity and specificity, receiver operating curves and the area under the curve (AUC).

**Results:**

The five-category model had overall accuracy of 82.9% in the validation dataset, a sensitivity of 0.681 for entering a net, 0.632 for exiting, 0.733 for net down, and 0.800 for net up. A simplified four-category model, combining entering/exiting a net into one category had accuracy of 94.8%, and increased sensitivity for net down (0.756) and net up (0.829). A further simplified three-category model, identifying sleeping, net up, and a combined net down/enter/exit category had accuracy of 96.2% (483/502), with an AUC of 0.997 for net down and 0.987 for net up. Models for detecting entering/exiting by adults were significantly more accurate than for children (87.8% vs 70.0%; p < 0.001) and had a higher AUC (p = 0.03).

**Conclusions:**

Understanding how LLINs are used is crucial for planning malaria prevention programmes. Accelerometer-based systems provide a promising new methodology for studying LLIN use. Further work exploring accelerometer placement, frequency of measurements and other machine learning approaches could make these methods even more accurate in the future.

**Supplementary Information:**

The online version contains supplementary material available at 10.1186/s12936-022-04102-z.

## Background

The distribution of long-lasting insecticidal bed nets (LLINs) is one of the main malaria control strategies in malaria-endemic countries. The World Health Organization (WHO) has called for universal access to LLINs for each of the over 3 billion people worldwide at risk of malaria [1]. Household LLIN ownership is associated with an 18–23% reduction in all-cause child mortality [2] and LLINs accounted for an estimated 68% of the 40% reduction in malaria incidence between 2000 and 2015 [3]. Since 2015, however, progress in malaria control has plateaued. In 2019, there were an estimated 409,000 deaths due to malaria, despite 253 million LLINs being delivered to endemic countries [4]. The distribution of LLINs is increasingly seen as insufficient on its own to re-ignite progress towards reducing the significant burden of malaria for the world’s poorest people.

A crucial question in evaluating future priorities for national malaria control programmes (NMCPs) is understanding usage patterns in households that receive LLINs after universal distribution. Whether the continuing effectiveness of LLINs for malaria prevention hinges on evolving mosquito pyrethroid resistance [5, 6], shifts in vector-biting behaviour [7, 8], net durability [9], increasing access [10, 11] or other factors, accurate and precise measures of usage patterns are essential for planning the future role that LLINs should play in malaria prevention. The most common approach to measure LLIN use involves a series of questions in a household survey on whether LLINs were used the night before and, if so, by which household member(s). While responses to this question provide estimates of use at a specific point in time, they do not capture temporal patterns of use, such as time spent under a net throughout the night or seasonal variations in use, all of which can impact the level of protection provided [12]. Further, this method is subject to recall and social desirability biases that may result in inaccurate conclusions when characterizing bed net use in some contexts [13]. Night-time observations have been used in several settings to provide more detailed information on night-time activity and net use patterns but can be time and resource intensive [14]. Improved methodologies that can efficiently measure net use more granularly and over longer time scales could facilitate broader availability of actionable data for operational programmes, particularly for comparison against vector-biting behaviours and resulting malaria risk.

To address these challenges, new remote adherence monitoring tools have been deployed that can produce objective records of LLIN use over weeks at a time [15, 16]. These devices have been demonstrated to be acceptable to local populations [17] and are providing novel insights into LLIN-use behaviours in endemic countries [18]. Nevertheless, current approaches have been limited to tracking the time and date when an LLIN is unfurled for use or folded up for storage above a sleeping area. Adding to a prior study of commercially available accelerometers for measuring LLIN use [15], the goal of this study was to assess the performance characteristics of an accelerometer-based approach for non-obtrusive measures of a range of LLIN use behaviours in a controlled setting.

Here, machine learning tools were utilized in a proof-of-concept study to distinguish between five key behaviours with implications for malaria risk: (1) unfurling a net; (2) entering an unfurled net; (3) lying still as if sleeping; (4) exiting from under a net; and, (5) folding up a net. The hope is to establish a new, more granular, method for studying how and when LLINs are used in real-world conditions. This will improve the understanding of LLIN effectiveness and inform future priorities for the many prevention programmes throughout the world that rely on LLINs as the backbone of their malaria prevention strategy.

## Methods

### Study location, laboratory set-up and device configuration

**Fig. 1:**
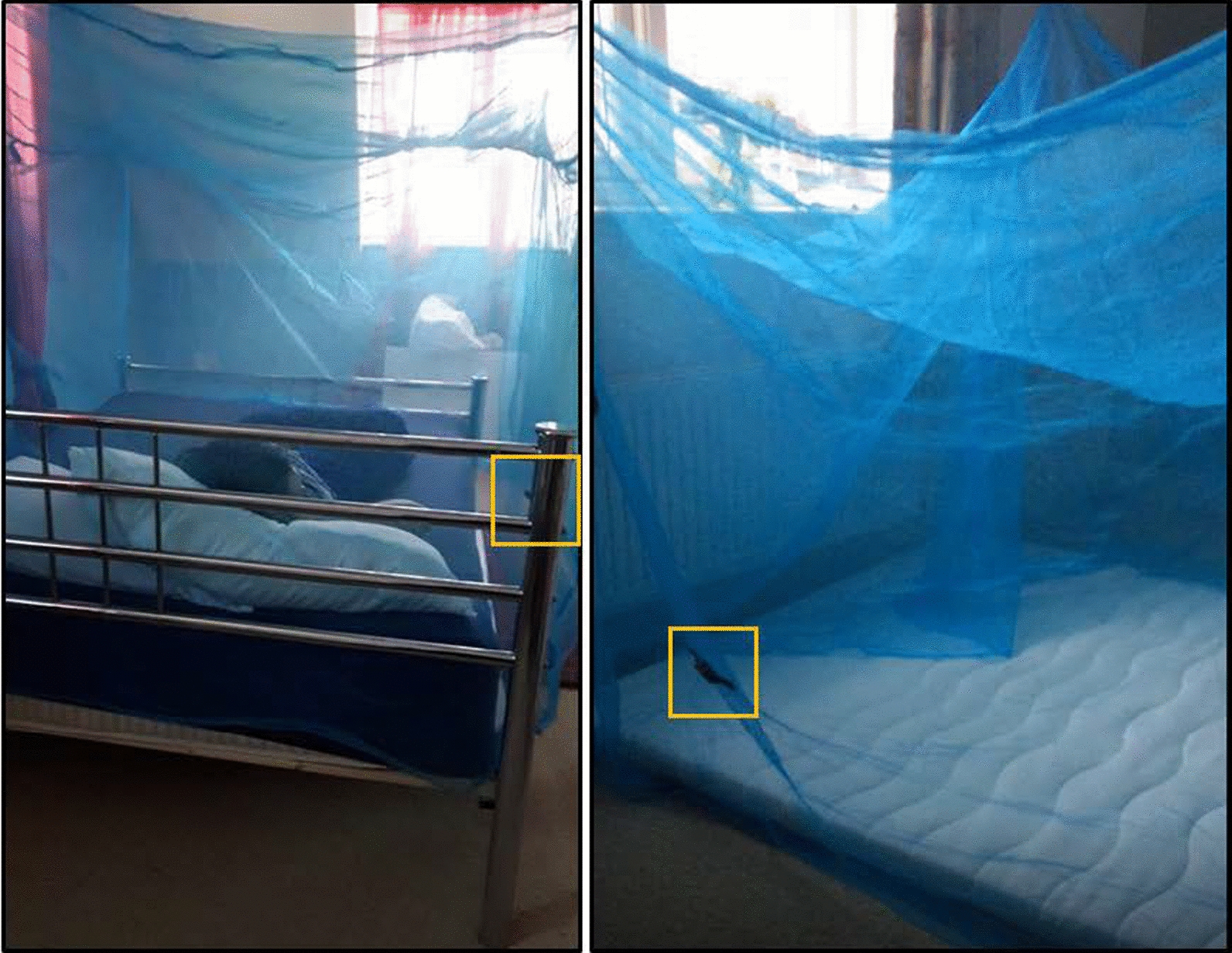
Photographs of accelerometer and placement when fixed on bed nets. Boxes identify where on the bed nets the accelerometers were fixed. In the photograph on the left, the device is visible just behind the bed post

This study was carried out in a furnished house in Liverpool, UK, under controlled conditions intended to mimic the ways that nets are typically hung, used and entered into/exited from in a malaria-endemic community. The study was carried out in two separate bedrooms from May to July 2018. One bedroom had a sleeping mat and the other bedroom had a bed with a mattress. The data were pooled for this analysis because there was no significant difference in the results between the sleeping surfaces. Each sleeping area had a standard rectangular net (190 cm long × 180 cm wide × 150 cm high) that was purchased in a market in Cote d’Ivoire. The bed nets were attached from their four corners to the ceiling above the sleeping area. The accelerometers used in the study were GENEActiv (ActiveInsights, UK) wrist-worn sensors, with the straps removed. One device was fixed 25 cm up from the bottom in the midline on one of the long sides of each net (Fig. 1). The accelerometer was placed on the side where the participant was instructed to enter/exit from the net. Before starting data collection, the accelerometers were calibrated, synchronized in time and set to collect data over 10-s epochs at a frequency of 10 Hz (10 readings per second).

### Sample size and participant recruitment

Based on experience from a previous study of accelerometers [15], a sample size of at least 25 individuals was targeted to reflect the variation of net-use actions, including both adults/children and men/women to evaluate the impact of individual size on accelerometer accuracy. This sample size represented a conservative estimate, informed by previous experience working with an older version of the accelerometers. Families were identified in the local community and approached for participation by one of the study authors (GBK). After explaining to the selected families the purpose, risk and benefits of being part of the study, prospective participants were shown examples of the low-resolution images (144 × 24 pixels) that would be used in the study as verification of the bed net use. Adult participants provided written informed consent and children provided assent in the presence of their parents or legal guardians. Transportation was provided each day from participants’ homes to the study and a return home in the evening. A small stipend was provided for each day of participation to compensate participants for their time.

### Capturing data from videos and accelerometers

After the participants consented and the sensors were attached to the nets, the participants were asked to mimic five basic motions: (1) unfurling a folded-up net (net down); (2) entering an unfurled net; (3) lying still as if sleeping under an unfurled net for 1 min; (4) exiting from under an unfurled net; and, (5) folding up a net (net up). An assistant with a low-resolution camera was trained to film each motion to ensure that the timing and format of the motions were synchronized between the sensors and the motions. When each participant made a motion, the motion type was recorded in a notebook with the start and end time for completing the motion. The computer clock used to initialize/configure the device was used as the reference time for the motions. Participants were asked to mimic the five motions 14–15 times each, with a 1-min break in between. Of the five basic motions listed above, children were only asked to perform the entering (No. 2), sleeping (No. 3) and exiting (No. 4) motions (due to inability to reach high enough for net up and net down motions). Children performed all of their activities in 1 day, but some adults returned to perform the activities over 2–3 days depending on availability. Additional measurements of the accelerometer were obtained when no participants were present with the net folded up and the net unfurled. However, because these data were indistinguishable from the sleeping data gathered when the participants were lying still for a few minutes as above, they were pooled together in the final analysis and are referred to subsequently as ‘sleeping/no activity’.

### Data extraction and device maintenance

Data extraction was done daily. The files were downloaded from the devices to *.bin* files and converted using the GENEActiv software to *.csv* format. The raw (*.bin*) data files ranged in size from 24 mb to 125 kb. Depending on the size of the data files, it took between 4 min and 30 s to download the data. In addition, at multiple points during the study, the times recorded by the observer were verified to match with those displayed by the digital camera and the computer. The notebook record with the motion type, start time and end time was recorded and applied to the corresponding observation from the accelerometer data. According to the manufacturers of the GENEActiv, the devices are capable of performing measurements at 10 Hz for 45 days on a single charge. To be conservative, the two devices were recharged once each during the study, after 2 weeks, for 4 h.

### Statistical analysis

Each data element for the analysis consisted of the observed record (tag) for the motion type applied to 20 s of accelerometer data. This was comprised of two 10-s time periods, with the average of the x, y and z values recorded 10 times per second over 10 s. This provides two x, y and z readings each per motion. In addition to the x, y and z dimension values, additional features were added to the data prospectively to increase the likelihood of identifying the motions of interest and classifying between them based on the distribution over time. These included, for each 10-s period, the standard deviations of each of the x, y and z dimensions and a value representing the sum of the magnitude of displacements in x, y and z dimensions. Overall, there were a total of 10 available features per observation.

As mentioned above, due to there being no significant difference in the results, data were pooled for the analysis making no distinctions between data from (1) the bed versus the sleeping mat; (2) no participant present versus a participant lying still under the net; and, (3) differences between men and women using the net. Thus, three separate analyses were performed. The first analysis included all observations for the recorded motions pooled together, regardless of whether the bed net was used by an adult or a child. The second analysis included only children and was restricted to the three activities performed by the children (sleeping/no activity, entering and exiting). The third analysis included only adults and was similarly restricted, for comparison, to the three activities performed by the children.

Due to observed challenges in distinguishing between the entering and exiting motions, three separate classification algorithms were trained: (1) a three-category classification of sleeping/no activity, net up or net down (with net down encompassing also both entering and exiting, as these occur only when the net is unfurled); (2) a four-category classification of sleeping/no activity, net up, net down or entering/exiting combined; and, (3) a five-category classification of each motion separately classified. Data were exported into R from the *.csv* format and split into 80/20 training and validation datasets. The classification of the observations was performed in R (version 4.0.2) using a supervised non-linear algorithm via the randomForest package [19]. Parameters for the randomForest algorithm were set to include 1000 trees and 4 variables randomly sampled as candidates at each split (4 ≈ sqrt[10 available features]). The machine learning algorithms were trained on the training dataset and then the performance of the algorithms was assessed using each model’s prediction of the motion type in the validation dataset compared to the actual value. The specificity, sensitivity, accuracy, and receiver operating characteristic (ROC) curves, including area under the curve (AUC) with 95% confidence intervals, were calculated for each motion type. According to convention, AUC and accuracy values ≥ 0.900 were considered excellent, 0.800 to 0.899 good, 0.700–0.799 fair, and ≤ 0.600 poor. DeLong’s method, a non-parametric approach for comparing two ROC curves, was used to compare the performance of the two models that restricted to adults and children [20].

## Results

Of a total of 30 individuals in 19 families approached, 18 adults (8 women and 10 men; aged 18–48 years) and 9 children (4 girls and 5 boys; aged 6 to 14 years) were enrolled. A total of 2506 observations were included in the analysis, comprising 1214 (48.4%) sleeping/no activity, 461 (18.4%) entering, 462 (18.4%) exiting, 185 (7.4%) folding net up, and 184 (7.3%) unfolding net. The full dataset was then randomly split into 2004 (80%) observations for the training dataset and 502 (20%) for the validation dataset.

**Fig. 2:**
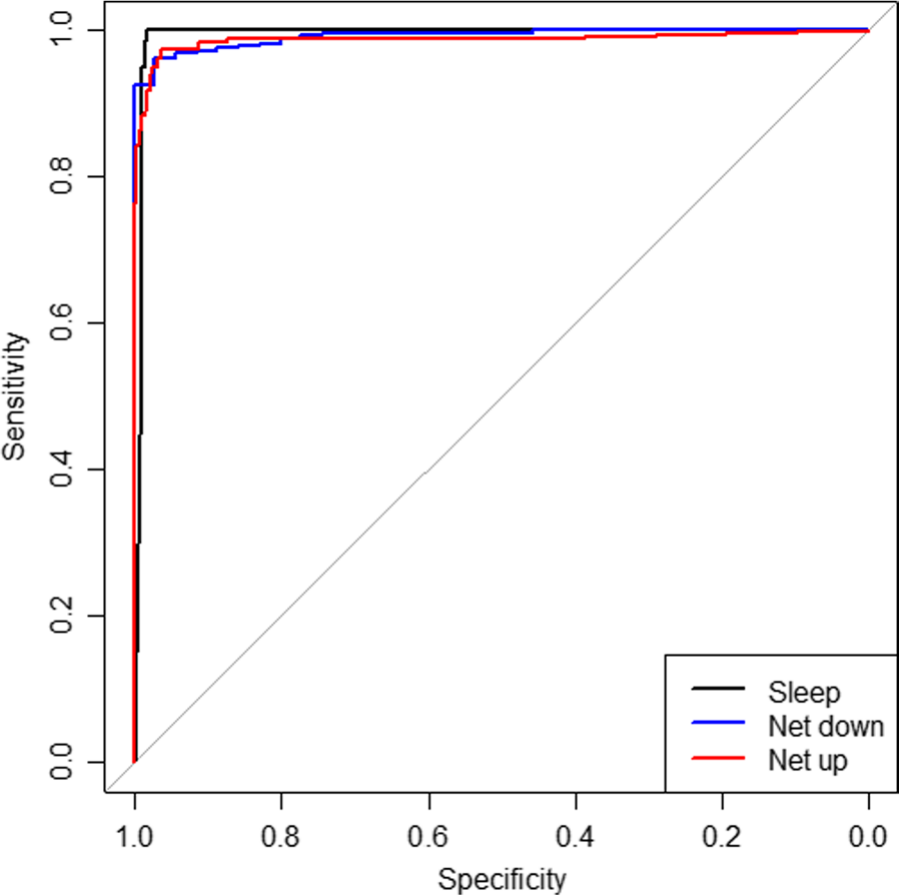
Receiver operating characteristics for the three-category classification model

The three-category model had an overall accuracy of 96.2% (483/502) in the validation dataset. It performed well in identifying the combined category of net down/enter/exit motions (AUC 0.9916 (0.985 to 0.998)) with excellent sensitivity (0.957) and specificity (0.966). The AUC for net-up motions was 0.987 (0.975 to 0.998) with excellent specificity (0.996), but sensitivity was only moderate at 0.771 (Table 1 and Fig. 2). The net-up motions were more frequently misclassified as a net-down motion (8 of 13 misclassifications) than sleeping/no activity (5 of 13) (Additional file 1: Table S1).

**Table 1:** Performance of three-category classification model for bed net use behaviours in validation dataset

Net use behaviour	Observations	Sensitivity	Specificity	AUC (95% CI)
Sleep/no activity	233	0.996	0.981	0.992 (0.983–1.000)
Net down/enter/exit^a^	254	0.957	0.966	0.992 (0.985–0.998)
Net folded up	35	0.771	0.989	0.987 (0.975–0.998)
Overall accuracy	483/502 (96.2%)			

*AUC* area under the curve, *CI* confidence interval

^a^Comprises activities that occur when net is in use: unfurling net and entering/exiting unfurled net

**Fig. 3:**
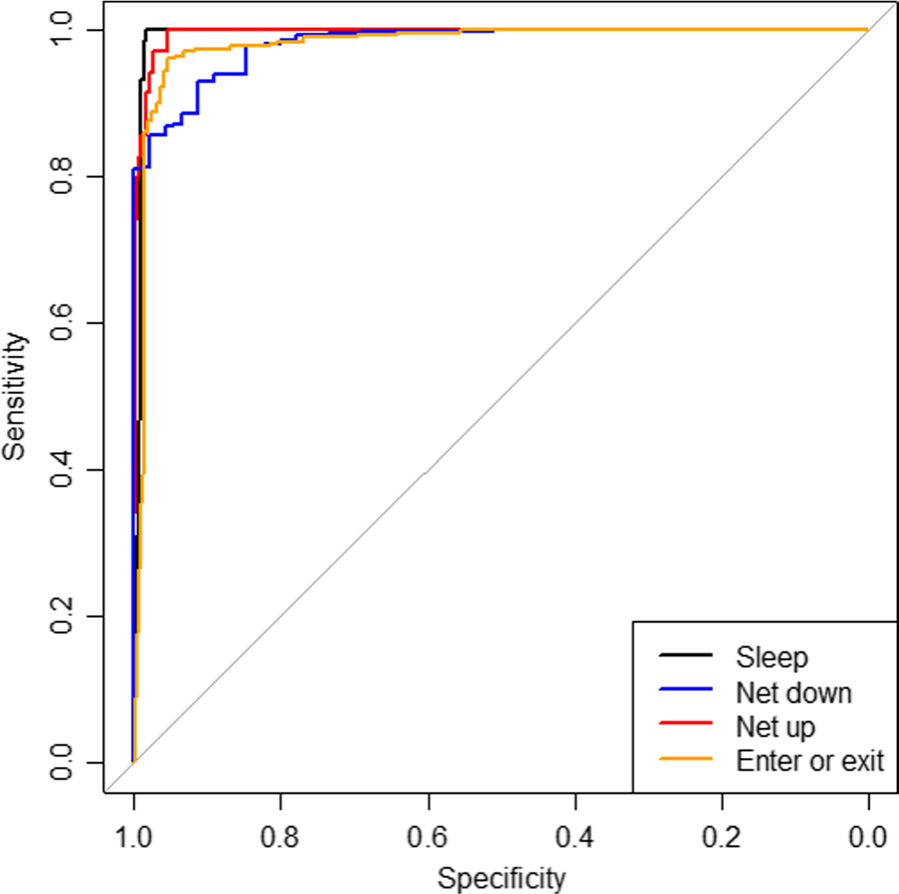
Receiver operating characteristics for the four-category classification model

In the four-category classification model, overall accuracy was nearly as high as the three-category model at 94.8% (476/502). Sleeping/no activity continued to be easily identifiable (AUC 0.992 (0.984 to 1.000)). Net-down motions (here restricted to unfurling a net with enter/exit having a separate category in this model) had an AUC of 0.981 (0.968 to 0.994) with moderate sensitivity of 0.756. Net-up motions had a similar AUC of 0.994 and a higher sensitivity of 0.829 than in the three-category model. The combined category for entering or exiting an unfolded net had an AUC of 0.979 (0.965 to 0.994) and excellent sensitivity (0.952) and specificity (0.952) (Table 2 and Fig. 3).

**Table 2:** Performance of four-category classification model for bed net use behaviours in validation dataset

Net use behaviour	Observations	Sensitivity	Specificity	AUC (95% CI)
Sleep/no activity	233	1.000	0.981	0.992 (0.984–1.000)
Net folded down	45	0.756	0.996	0.981 (0.968–0.994)
Net folded up	35	0.829	0.991	0.994 (0.989–0.999)
Enter or exit net	189	0.952	0.952	0.979 (0.965–0.994)
Overall accuracy	476/502 (94.8%)			

*AUC* area under the curve, *CI* confidence interval

**Fig. 4:**
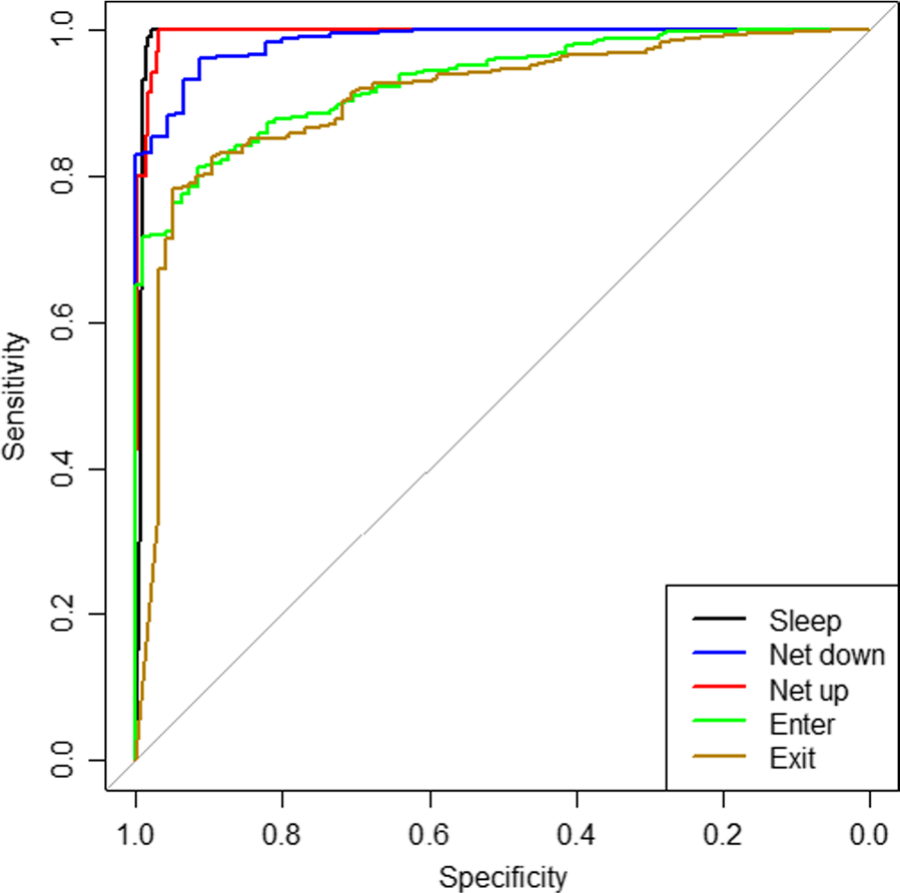
Receiver operating characteristics for the five-category classification model

In the full five-category classification model, overall accuracy decreased but was still good at 82.4% (416/502). Sleeping/no activity continued to be identified well (AUC 0.993). The AUC was also excellent for each of the four other categories, including 0.985 (0.973–0.996) for net down, 0.994 (0.989 to 0.999) for net up, and 0.930 (0.908 to 0.952) and 0.906 (0.872 to 0.940) for entering and exiting a net, respectively. The specificity remained good to excellent for all five categories. The sensitivity was markedly lower, however, for identifying entering (0.681) and exiting a net (0.632) (Table 3 and Fig. 4). In the confusion matrix (Additional file 1: Table S2), entering and exiting were most commonly confused with each other, representing 83.3% (25/30) and 85.7% (30/35) of the classification errors, respectively.

**Table 3:** Performance of five-category classification model for bed net use in validation dataset

Net use behaviour	Observations	Sensitivity	Specificity	AUC (95% CI)
Sleep/no activity	233	0.991	0.981	0.993 (0.984–1.000)
Net folded down	45	0.733	0.989	0.985 (0.973–0.996)
Net folded up	35	0.800	0.989	0.994 (0.989–0.999)
Enter net	94	0.681	0.917	0.930 (0.908–0.952)
Exit net	95	0.632	0.909	0.906 (0.872–0.940)
Overall accuracy	416/502 (82.9%)			

*AUC* area under the curve, *CI* confidence interval

An importance plot was produced (Additional file 1: Fig. S1), to identify which of the variables in the classification trees made the most difference in increasing classification accuracy. Across all three models, although not always in the same order, the top three variables were the average over the y dimensions in the first and second 10-s periods and the standard deviation of the first epoch in the y dimension. Not surprisingly, this suggests that early up and down motions of the accelerometer are especially important in distinguishing between the net use behaviours.

For the comparison of adults and children, separate models were run for both by restricting the data to the three motions that the children performed: entering an unfurled net; sleeping/no activity; and, exiting a net. Comparing these models demonstrates that the model was more accurate for adults compared to children (87.8% vs 70.0%; p < 0.001). The models identified sleeping/no activity well for both adults and children (AUC of 0.994 vs 0.999; p = 0.323), but there were significant differences favouring the adult models when comparing the AUC for entering (0.934 vs 0.798; p = 0.03) and exiting a net (0.927 vs 0.800; p = 0.03) (Table 4).

**Table 4:** Performance of three-category classification model for bed net use behaviours in validation dataset

Net use behaviour	Adults				Children				Comparison of AUC
	Observations	Sensitivity	Specificity	AUC (95% CI)	Observations	Sensitivity	Specificity	AUC (95% CI)	
Sleep	1115	0.995	0.967	0.994 (0.985–1.000)	99	1.000	0.951	0.999 (0.995–1.000)	p = 0.323
Enter net	362	0.636	0.946	0.934 (0.907–0.962)	99	0.556	0.810	0.798 (0.684–0.911)	p = 0.026*
Exit net	363	0.730	0.902	0.927 (0.900–0.953)	99	0.565	0.784	0.800 (0.689–0.909)	p = 0.030*
		Overall accuracy		318/368 (86.4%)		Overall accuracy		42/60 (70.0%)	

*AUC* area under the curve, *CI* confidence interval

## Discussion

This study adds to a previous study of the use of commercially available accelerometers for the detection of bed net use [15] to demonstrate, in a controlled trial, that accelerometers can accurately classify real-world bed net use behaviours remotely over weeks at a time. The system, aided by machine learning based on a robust classification tree algorithm, was able to distinguish with high accuracy when bed nets were unfurled and folded up. The algorithm also performed moderately well in the most complex classification, achieving 83% accuracy in identifying exiting and entering an unfurled bed net, in addition to basic net up or net down use behaviours. Overall, combining the robustness, battery performance and the classification performance demonstrated here, accelerometer-based systems provide a promising new methodology for identifying with more granularity the temporal patterns of LLIN use in malaria-endemic regions.

The challenges of the current standard measurement tool, self-reported use the previous night, are well documented and render self-reported bed net use a reasonable measure for widespread surveillance, but a more limited tool for measuring more nuanced bed net use behaviours [21, 22]. The accuracy of self-reported LLIN use is constrained by potential biases and limitations, including: (a) social desirability bias; (b) recall bias; and, (c) sampling bias related to queries focused on a single night which may miss important seasonal and other temporal variations in use [12, 23]. Additionally, in most countries, surveillance with self-reported measures from household surveys occurs less than once per year, making monitoring trends on even an annual basis challenging. Individual patterns of use and trends in use over time have crucial importance for understanding malaria risk [24]. Remote objective monitors help mitigate many of the biases inherent in current bed net use surveillance and can elucidate net use patterns that would otherwise be infeasible without direct visual monitoring [18]. While these accelerometers have been found to be generally acceptable to local communities [17], future work will need to establish whether bed net use behaviours are affected by the monitoring itself.

One of the important findings of this study is the capability of the simple, unobtrusive accelerometers to identify more complex bed net use behaviours than have previously been possible. Both the three- and four-category classification models achieved excellent accuracy of 95% or greater and provide reliable data about whether the bed net is folded up (providing no protection) or unfurled. The high accuracy of the four-category model was encouraging. While this model did not distinguish between entering and exiting a net, it could still add significant insight into the patterns of bed net use as they relate to individual movements during the night. Such patterns may play a significant role in individual residual malaria risk notwithstanding the high bed net use rates reported with current surveillance methods. With lower accuracy and sacrificing some sensitivity, the five-category model was able to distinguish moderately well between entering and exiting a net. Not surprisingly, the models that sought to classify entering and exiting were more accurate among adults, who presumably triggered an easier-to-identify signature given their larger size than children. All of the models here, even the lowest accuracy but highest complexity, may arguably be a more realistic measure of actual bed net use behaviours than self-reports. Indeed, the measurement error inherent in the lower sensitivity categories in this study is on par with another study that attempted to quantify the bias in self-reports by comparing self-reported and objective measures [13].

Furthermore, there is reason to expect that the machine learning approaches used to perform the classification tasks in this study can be improved in multiple different ways. First, this study provides insights into changes that may improve the current method in future applications. These insights include the identification of the first 10 s as the most important in the classification, simplifying the data gathering and signal identification. In future work, it would be valuable to explore whether shorter periods would result in higher accuracy. It was also clear that the y dimension (vertical axis) is a crucial component in the classification system and may help to simplify future analyses. Second, random forest classification trees are only one of many machine learning methods that might be suitable in this setting. Neural networks, in particular, may be a promising avenue for improving classification accuracies. Third, the devices in this study were set to gather data 10 times per second, but more frequent sampling could identify the more nuanced behaviours, such as entering and exiting a net, at a higher accuracy. GENEActiv devices can gather data up to 1000 times per minute with an estimated 12-h battery life. Optimizing the trade-off between battery life and sampling frequency will be an important area for future work. Finally, each of the net use behaviours here were classified independently of each other. In real life, there are logical and timing constraints that can be utilized in an iterative fashion and leveraging model confidence to improve accuracy. For example, it is not possible to unfurl a bed net twice in succession. Thus, if the system identifies with high probability that a net has been unfurled, that information can be used to improve the accuracy of the next classification. These are all avenues for improvements in future work to create an even more accurate and flexible system.

Remote adherence monitors will improve our understanding of how LLINs are used relative to malaria risk, sociodemographic factors, vector abundance, and various climate variables such as temperature and humidity. Many studies have identified characteristics associated with poor bed net use by self-reports, but there remains a lack of precision in these measures to correlate patterns of bed net use with patterns of malaria risk that may vary season to season, month to month and even hour to hour [25]. Quantifying bed net use with more granularity allows for deeper inquiries into how LLIN use fits into the daily lives of those at risk of malaria. This may allow us to identify groups of people at high risk of malaria and reveal the need for complementary malaria control interventions in addition to LLINs. Identifying high-risk groups relative to their use of LLINs, which remain the most prevalent malaria prevention tool in the world, can also help maximize the efficiency of limited health resources by promoting better and more highly targeted behaviour change and malaria prevention programmes.

This study has clear limitations beyond those specific to the measurement tool and analysis discussed above. First, the study took place in a controlled setting in a non-malaria-endemic region. While the study attempted to mimic real-life bed net use, it is plausible that the motions of bed net users in actual practice might be different than in this study, although significant differences, at least in unfurling and folding up a net, seem unlikely. Other environmental characteristics surrounding real-world use of bed nets, on the other hand, are likely to be markedly different than those in this controlled experiment, such as the number of net users, the type of sleeping space, environmental (temperature, humidity, etc.) and other ‘noise’ factors (such as shaking of the net in a household environment due to other family members, domestic animals, or wind). There is evidence that the accelerometer approach may be robust to some of these variations, since the method was unaffected by whether the net was placed over a bed or a mat, for example. But the difference in performance in measuring the entering and exiting of children versus adults suggest that these noise factors deserve be explored more completely in future work, perhaps with more frequent measurements in Hz and at increased granularity (compared to the 20-s periods used here). Second, in this study the accelerometer was positioned on only one side of the net, which makes the entering and exiting data only relevant for entering and exiting from that side. Future work will need to explore whether two accelerometers will be necessary on both sides to capture accurate entering/exiting data, or whether an accelerometer can be placed on top of the net and still accurately obtain relevant data. Third, assessing each tagged motion as separate from each other as in this study raises the question of how to manage the data that will arrive from the field in one continuous stream. In future work, the classification system will need to identify, first, whether an ‘event’ has occurred from continuous data and, second, make an estimate as to the type of that event. It is likely that this will be a manageable challenge, given the ability of the system to identify at very high accuracy sleeping/no activity. Fourth, despite evidence showing the acceptability of remote adherence monitoring of bed net use in research settings [17], communication with local communities about what exactly is being monitored will be essential to ensuring continued acceptability. Finally, there are limitations to the information that this technology can provide. Additional work will be needed to link LLIN use data with specific individuals in a household. Further, net use patterns within the home must be paired with an understanding of broader night-time activity, movement patterns, and vector-biting behaviour for a more complete picture of when and where gaps in protection arise.

## Conclusion

Beyond the coverage attained through LLIN distributions, how LLINs are used is a crucial bedrock of malaria prevention. An LLIN unused or used incorrectly does not prevent malaria, particularly in the era of pyrethroid resistance and waning community effect [26, 27]. A more detailed understanding of sleep timing and timing of potential exposure to vectors among the millions of LLIN users worldwide may have relevance for malaria prevention on a broad scale, particularly considering the large share of malaria expenditure that goes toward LLINs [4]. Accelerometer approaches to remotely measure LLIN use represent an exciting new opportunity to understand malaria transmission and prevention as they intersect with human behaviour. This will improve our understanding of LLIN effectiveness and help improve malaria prevention where LLINs are relied upon as the backbone of malaria prevention strategies.

## Supplementary Information

**Additional file 1.** Confusion matrices and importance plot. Additional data to aid in the interpretation of random forest classifications.

## Data Availability

The datasets used and/or analysed during the current study are available from the corresponding author on reasonable request.
