# Supplementary material for: Evaluation of an accelerometer-based monitor for detecting bed net use and human entry/exit using a machine learning algorithm
Source: Malar J. 2022 Mar 12;21:85. doi: 10.1186/s12936-022-04102-z (PMC8917707; doi:10.1186/s12936-022-04102-z)

| Table S1: 3 category confusion matrix | | | |
| --- | --- | --- | --- |
|  | **Predictions** |  |  |
| **Observed** | Sleep | Net down | Net up |
| Sleep | 232 | 0 | 1 |
| Net down | 0 | 27 | 8 |
| Net up | 5 | 5 | 224 |
|  |  |  |  |

Additional file 1

| Table S2: 5 category confusion matrix | | | |  |  |
| --- | --- | --- | --- | --- | --- |
|  | **Predictions** |  |  |  |  |
| **Observed** | Sleep | Enter | Exit | Net up | Net down |
| Sleep | 231 | 1 | 1 | 0 | 0 |
| Enter | 1 | 64 | 25 | 3 | 1 |
| Exit | 3 | 30 | 60 | 2 | 0 |
| Net up | 0 | 2 | 28 | 28 | 4 |
| Net down | 1 | 1 | 0 | 0 | 33 |

Fig. S1: 5 category importance plot


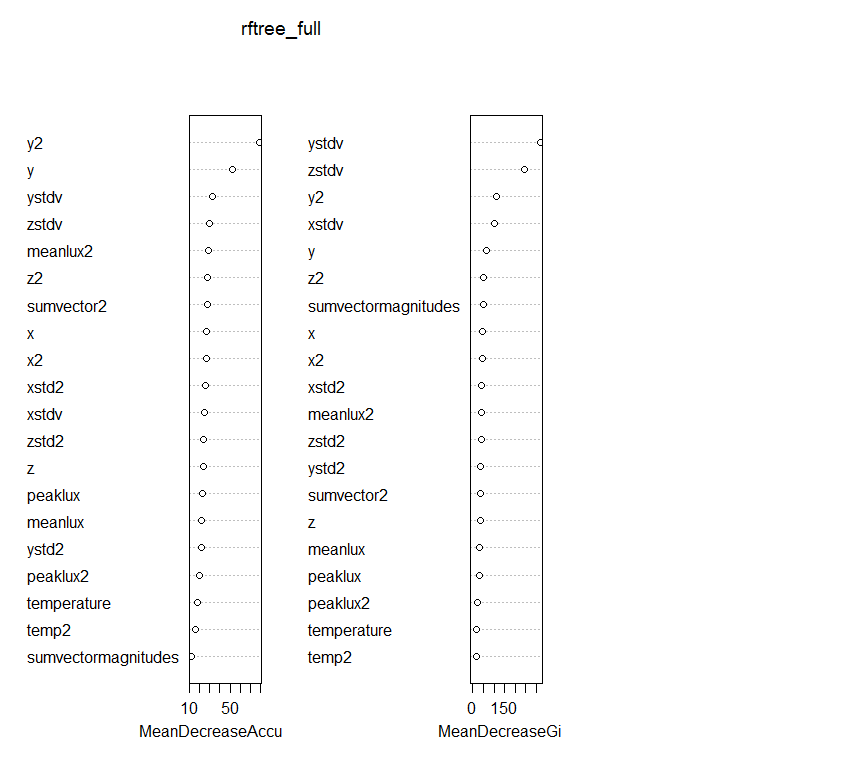

Supplement: Supplementary file 1 — Additional file 1. Confusion matrices and importance plot. Additional data to aid in the interpretation of random forest classifications. [file 12936_2022_4102_MOESM1_ESM.docx]
